# Factors to Consider in the Association Between Soy Isoflavone Intake and Breast Cancer Risk

**DOI:** 10.2188/jea.JE20090181

**Published:** 2010-03-05

**Authors:** Chisato Nagata

**Affiliations:** Department of Epidemiology & Preventive Medicine, Gifu University Graduate School of Medicine, Gifu, Japan

**Keywords:** breast cancer, soy isoflavones, review

## Abstract

It has been suggested that soy isoflavones have protective effects against breast cancer. However, data from epidemiological studies are not conclusive. A recent meta-analysis showed that soy intake was inversely associated with breast cancer risk in Asian but not Western populations, which indicates that protection against breast cancer may require that women consume levels of soy typical in Asian diets. In addition to the amount of soy isoflavones consumed, the form and food source of isoflavones, timing of isoflavone exposure, estrogen receptor status of tumors, and equol-producer status and hormonal profile of individuals may modify the association between soy isoflavone intake and the risk of breast cancer. These factors might explain the heterogeneity of results from studies. This present report contrasts background data from Japanese and Western women to identify the potential modifying of these factors.

## INTRODUCTION

There has been much interest in the potentially protective role of soy in breast cancer development. Soybeans are the main source of isoflavones, which are classified as phytoestrogens. Laboratory data show that isoflavones have a wide range of biological actions. First, they have an affinity for estrogen receptors in vitro.^1^ Thus, they may act as antiestrogens by competing for the binding sites of estrogen receptors. Isoflavones also inhibit the activity of key enzymes that convert androgens to estrogens.^2^ In addition, isoflavones have been shown to be anti-proliferative,^3^ proapoptic,^4^ anti-angiogenic,^5^ anti-oxidative,^6^ and anti-inflammatory.^7^ Taken together, these findings indicate that soy isoflavone may be a potent agent for preventing breast cancer. However, some data suggest that isoflavones promote breast cancer. Indeed, it has been shown that genistein stimulates the growth of estrogen-sensitive breast cancer cells in ovariectomized mice.^8^^,^^9^ Because of the apparent complexity of the relationships between isoflavones and breast cancer in laboratory studies, data from human studies are particularly important. However, current data from epidemiological studies are not conclusive. There is some epidemiological evidence of an inverse association between isoflavone and breast cancer, but it is inconsistent.^10^ Thus, no consensus has emerged regarding the preventive aspects of isoflavones.

Recently, Wu et al conducted 2 separate meta-analyses of studies carried out in Asian and Western populations.^11^ A meta-analysis of 8 studies showed that Asian women consuming the highest amount (≥20 mg/day) of dietary isoflavones had a 29% reduction in breast cancer risk, as compared with those with low consumption (<5 mg/day) of isoflavones. An approximately 50% reduction in risk was reported in a prospective study of Japanese women.^12^ In contrast, a meta-analysis of 11 studies of women eating Western diets found no association between isoflavone intake and breast cancer risk. In that analysis, the median for the highest intake was 0.8 mg/day. Therefore, women may need to consume the amount of soy in Asian diets to gain protection against breast cancer. Alternatively, the protective effects may only be present when exposure to soy occurs early in life. Differences in (1) the form and source of isoflavones, (2) the biological response to isoflavones among ethnic groups, and (3) the interactions with individuals’ hormonal profiles may also explain the heterogeneity of the findings. This present report attempts to address these issues by contrasting background data from Japanese and Western women.

## SOY ISOFLAVONE INTAKE IN JAPAN

The incidence rate of breast cancer is historically much lower in Japan than in Western countries. One possible explanation for this is that Japanese consume much more soy foods containing isoflavones. The fact that the incidence rate of breast cancer among Japanese immigrants rises as the length of time in the host country increases^13^^,^^14^ suggests that lifestyle changes, including a change in dietary isoflavone intake, may play a role. According to the Cancer Incidence in Five Continents Vol IX,^15^ the incidence rate for breast cancer in 1998–2002 was 32.0 per 100 000 among Japanese women in Osaka, Japan; 105.6 per 100 000 among white women in Hawaii; and 107.5 per 100 000 among Japanese women in Hawaii.

The nutritional status of Japanese has been evaluated annually by the National Nutritional Survey (NNS)^16^ for more than 50 years. About 20 000 individuals from 6000 households in a randomly selected 300 districts are invited to participate in this survey every year. Foods and beverages consumed by a family are weighted and recorded for 3 consecutive days (1-day records have been used since 1995). According to the NNS reports, the mean intake of soy products was 67.2 g/day in 1975 and 57.7 g/day in 2005. Recently, the Food Safety Commission in Japan estimated isoflavone intake (expressed as aglycone) among Japanese, using the 2002 NNS data (response rate unknown). The median and the 95th percentile isoflavone intakes were 18 and 70 mg/day, respectively. Mean estimates of daily consumption reported in other studies in Japan ranged from 26 to 54 mg.^17^ A database of the isoflavone content of Japanese foods, based on a validated direct measurement method, was first reported by Kimira et al.^18^ In a report from the Takayama study—a prospective study of residents in Takayama city, Gifu, Japan^19^—the mean estimate, based on a validated food frequency questionnaire, was 19.6 mg per 1000 kcal among 15 724 female residents aged 35 years or older (response rate, 85.3%).^20^ Daily intake of isoflavones is estimated to be between 0.5 and 3 mg in the United States^21^^,^^22^ and less than 2 mg in European countries.^23^ In a recent study of 8809 adults from the National Health and Nutrition Examination Survey (NHANES), the mean isoflavone intake, based on one 24-hr diet recall, was 1.2 mg.^24^ Changing dietary habits have been reported in migrant populations. For example, soy intake among Japanese-Americans appears to be higher than that of US whites and lower than that of Japanese living in Japan. Takata et al^25^ reported that mean isoflavone intake, estimated from a food frequency questionnaire, was 11.6, 7.2, and 3.5 mg per 1000 kcal in Japanese women in Gifu, and Japanese and white women living in Hawaii, respectively. According to the 2002 NNS data in Japan, isoflavone intake was associated with age. Even among adults, younger age groups tended to consume less isoflavones: the reported mean daily intake of isoflavones was 19.4, 21.5, 23.5, 27.4, 33.6, and 29.6 mg per day for adults aged 20–29, 30–39, 40–49, 50–59, 60–69, and 70+ years, respectively. Soy isoflavone intake will likely decrease among Japanese in the future.

Biomarkers are also useful in estimating exposure to soy isoflavones. Yamamoto et al^26^ collected 24-hour urine and serum samples from Japanese women enrolled in the Japan Public Health Center-based (JPHC) prospective study. The mean levels for daidzein and genistein were 17.0 (standard deviation [SD], 15.3) and 14.2 (14.1) µmol/day, respectively, in urine. The corresponding values in plasma were 119.9 (135.8) and 475.3 (510.4) nmol/L, respectively. These values were reasonably correlated with isoflavone intake estimated from food-frequency questionnaires or diet records (*r* = 0.22–0.48). Similar values in the urine, plasma, and serum of Japanese women have been reported in other studies.^27^^–^^29^ Even in spot urine samples, isoflavone levels were well correlated with intakes estimated from diet records (*r* = 0.30 for daidzein and 0.27 for genistein).^30^ In a study of 59 women aged 20 to 45 years, the median values were 4.0 and 3.2 nmol/mg creatinine for daidzein and for genistein, respectively, in first morning urine specimens.^31^ Another study of 419 women reported that the mean daidzein level was 12.8 nmol/mg creatinine in spot urine samples collected at approximately 2:00 PM.^32^ Among Western populations, reported urinary isoflavone levels have been much lower. The geometric mean levels were 0.27 and 0.08 nmol/mg creatinine for daidzein and genistein, respectively, in urine specimens of participants in the NHANES between 1999 and 2000.^33^ In a study of UK women, the geometric means for daidzein and genistein were 0.55 and 0.28 nmol/mg creatinine, respectively, in spot urine, and 7.9 and 15.2 µmol/L, respectively, in serum.^34^

Sources of isoflavones also differ between Japanese and Western populations. The intake of traditional soy-based foods is high in Japan. Based on data from the Takayama study, the most common soy foods are tofu, miso (soybean paste), soybeans, fried tofu, natto (fermented soybeans), and soymilk. The fermented soy foods (miso and natto) accounted for about 40% of total isoflavone intake. In Western populations, the consumption of traditional soy foods is substantially lower, and a portion of total isoflavone consumption is derived from soy protein and soy flour added to a variety of foods.^22^ Such uses are increasing.^35^ Recently, new isoflavone datasets for non-soy based foods have been developed to estimate isoflavone intake more accurately. Horn-Ross et al^22^ assessed the isoflavone intake in 447 non-Asian women in the San Francisco Bay area, using a new database. Mean intakes of genistein and daidzein were estimated to be 1.5 and 1.3 mg/day, respectively. Major sources were tofu, doughnuts, soymilk, white bread, and canned tuna. Interestingly, doughnuts accounted for about 20% of the average of daily intake of genistein and 15% of daidzein intake. It is likely that Japanese women are also consuming isoflavones from such “hidden” sources of soy. Using several databases of non-soy foods, such as cereals, eggs, dairy foods, meats, fish, nuts, and vegetables,^21^^,^^36^^–^^39^ and assuming that processed meats and fish contain 2% soy protein by weight, isoflavone intake from these non-soy foods was estimated to be approximately 0.68 mg per 1000 kcal among the women in the Takayama study (unpublished data). This value is substantially lower than the isoflavone estimate from soy foods (19.6 mg per 1000 kcal).

In fermented soy products, like natto or tempeh, aglycones are the principal form of isoflavone, whereas in unfermented soy products, like soy milk or soy supplements, the glucoside form is predominant.^39^ Knowing the bioavailability of the different isoflavone forms is thus fundamental. Watanabe et al conducted a kinetic study of Japanese men after ingestion of 60 grams baked soybean powder (*kinako*).^40^ Differences in the form of isoflavone (aglycone vs glucoside) are believed to alter isoflavone pharmacokinetics and, hence, the association between soy intake and breast cancer risk. However, there is no consensus among studies on the bioavailability of aglycone vs glucoside.^41^ In addition to isoflavone form, the matrix in which it is delivered is likely to have an effect on pharmacokinetics. It is also possible that an individual’s background diet may modify the association of isoflavone intake with breast cancer risk by affecting the pharmacokinetics of metabolism of isoflavones. In addition, frequency of ingestion may affect bioavailability of isoflavones.^41^ An optimum steady-state serum isoflavone level would be expected from consuming relatively small doses of soy throughout the day, as opposed to a single dose at one time. Indeed, Gardner et al^42^ observed that plasma isoflavone concentrations remained constant when soy foods were consumed at breakfast, lunch, and dinner.

Metabolism of isoflavone can vary greatly between individuals, even when they consume an identical amount of isoflavone-containing food.^43^ Equol, a metabolite of daidzein, has been identified in the urine and blood samples of some but not all humans. Intestinal bacteria play an essential role in daidzein metabolism. Several candidate bacteria for daidzein metabolism, including one reported by Ueno et al,^44^ have been suggested, but the human intestinal bacteria responsible for daidzein metabolism are yet to be identified.^45^ Equol binds with greater affinity to estrogen receptors than its parent compound daidzein,^46^ and it is superior to all other isoflavones in its antioxidant activity,^47^ which suggests that its overall potency is greater. The prevalence of equol producers appears to be higher in Japanese than in whites,^27^^,^^48^^,^^49^ which might explain the additional benefits conferred on Japanese women in terms of reduced breast cancer risk.

## EARLY EXPOSURE TO SOY

In vivo studies have consistently shown that prepubertal or pubertal exposure to genistein reduces the incidence and/or multiplicity of chemically induced mammary tumors in experimental animals,^50^ possibly because of alterations in gene expression and morphological changes in the mammary glands. The details have been described elsewhere.^51^ To this author’s knowledge, 3 case-control studies have examined the association between soy intake during childhood or adolescence and the risk of breast cancer among Asian women.^52^^–^^54^ All reported a significant inverse association. Another case-control study of Canadian women also observed a significant inverse association between adolescent isoflavone intake and breast cancer risk.^55^ A recent prospective study of Chinese women confirmed that a high intake of soy foods during adolescence was associated with a reduced risk of breast cancer.^56^ Unfortunately, there has been no such study among Japanese women.

Perinatal factors are also thought to influence subsequent breast cancer development. Trichopoulous hypothesized that the developing breast is influenced by the fetal environment, particularly variations in hormone concentrations, which could mediate subsequent breast cancer development.^57^ Some epidemiological studies observed associations between pre- and perinatal characteristics, such as birth order and birth weight, and subsequent risk of breast cancer.^58^ There has been no study assessing the association between maternal soy intake during pregnancy and the risk of breast cancer in offspring; however, by altering the estrogen environment, soy exposure in utero may affect the subsequent risk of breast cancer.

In utero soy exposure occurs among Japanese. Adlercreutz et al^59^ reported that in 7 Japanese women at delivery the mean levels of genistein and daidzein were 83.9 and 45.5 nmol/L, respectively, in maternal plasma, and 165 and 58.8 nmol/L, respectively, in umbilical cord plasma. They observed high correlations between levels in maternal and umbilical plasma (*r* = 0.34 for genistein, *r* = 0.44 for daidzein, and *r* = 0.99 for equol; *n* = 7). A study by the present author, which included 194 pregnant women, supported the hypothesis that isoflavone can be transferred from mother to fetus.^30^ In that study, we also included an assessment of maternal dietary intake of soy during pregnancy and measurements of estrogen levels in maternal and umbilical cord blood; the mean soy isoflavone intake was 21.7 mg per day. The geometric mean levels for serum genistein and daidzein were 116.5 and 50.2 nmol/L, respectively, in maternal blood at delivery, and 126.9 and 38.6 nmol/L, respectively, in umbilical cord blood. Estradiol level in umbilical cord blood was unrelated to isoflavone levels in both maternal and umbilical cord blood.

Infants in Japan are weaned on to soy products between 6 and 12 months of age, after which they continue to receive isoflavone-containing foods indefinitely; tofu and miso soup are common baby foods. Based on intake frequencies of tofu, miso soup, natto, soybean flour, and soymilk reported by 288 mothers, 6-month-old Japanese infants consumed about 3.1 mg of soy isoflavone per day (unpublished data). Setchell et al^60^ reported that soy-based formulas contained approximately 32 to 47 µg/ml of isoflavone, indicating that a 4-month old infant fed soy formula would consume 28 to 47 mg of isoflavones per day. In their study, the mean plasma concentrations of genistein and daidzein were 2530 and 1160 nmol/L, respectively, in 7 infants fed soy-based formulas. An isoflavone intake of 3 mg for Japanese infants is not low, if we take into account the body size of babies; however, it would be much lower than that available from a soy-based formula. Badger et al^61^ reported substantial differences between exposure to soy between Japanese and Americans. If these differences are real, then Japanese fetuses would be exposed to the same high isoflavone levels that were present in maternal circulation. Serum isoflavone levels among Japanese infants would then be expected to decline at birth and remain low, until soy foods were introduced as baby food. Thereafter, the level would increase to the usual adult levels as a result of habitual intake. American infants fed soy formula would be expected to have high serum levels starting immediately after birth and persisting until they were weaned, at which time levels would drop and likely remain low thereafter.

## ESTROGEN RECEPTOR STATUS

Epidemiological studies have found that several hormone-related lifestyle factors, such as nulliparity, earlier age at menarche, higher body mass index (BMI), and use of postmenopausal hormones were related to elevated risk of estrogen receptor-positive, but not estrogen receptor-negative, cancers.^62^^,^^63^ Because isoflavones have an affinity for estrogen receptors, the protective effect of soy intake may be pronounced for estrogen receptor-positive tumors. To date, 6 studies have assessed the association of soy or isoflavone with breast cancer risk, with regard to the estrogen receptor status of tumors.^34^^,^^64^^–^^68^ Some of these studies, including 1 conducted in Japan, observed an inverse association of soy intake with the risk of estrogen-positive tumors,^64^^,^^65^^,^^67^ but not with the risk of other tumor types.^34^^,^^66^^,^^68^ The incidence rate of estrogen receptor-positive tumors is increasing in the United States.^69^ In addition, it has been reported that the prevalence of estrogen receptor-positive expression in normal breast tissue from Japanese women was lower than that in white women.^70^ This may simply be a result of the lower incidence of breast cancer among Japanese women. The determinants of estrogen receptor status in normal breast tissue and tumors are not known.

## HORMONAL PROFILE

It has been suggested that isoflavones have stimulatory effects in low-estrogen environments, and that in high-estrogen environments, they block the effects of estrogen.^71^ Evidence from case-control studies has supported a protective role for soy in premenopausal women versus postmenopausal women.^10^ The effects of soy and isoflavone on the level of circulating estrogen have been examined in many intervention studies. A recent meta-analysis of intervention studies revealed that although soy and isoflavone consumption did not affect estradiol or estrone, it did reduce FSH and LH in premenopausal women.^72^ Moreover, in postmenopausal women, soy and isoflavone were associated with a small but nonsignificant increase in total estradiol. These observations lend tentative support to the above hypothesis. However, in a cohort study that observed a significant inverse association between soy isoflavone intake and breast cancer risk among Japanese women, the association was somewhat stronger in postmenopausal women than in premenopausal women.^12^ A similar tendency was noted in another recent cohort study of Asian women.^68^ In addition, some studies have observed lower endogenous estradiol levels in low-risk populations than in high-risk populations.^73^^,^^74^ Although postmenopausal estrogen levels should not be compared when measured by different laboratories, estrogen levels appear to be low among postmenopausal Japanese women (ie, estradiol <6 pg/mL and estrone <10 pg/mL).^75^^–^^79^ Such a low-estrogen environment, which likely reflects low body fat mass, may favor the protective effects of soy on breast cancer in Japanese women. Some studies of soy and breast cancer risk included analyses stratified by BMI but not by estrogen level. The results were not consistent: one observed a somewhat stronger association in women with high BMI,^55^ but others noted no significant differences.^68^^,^^80^

## CONCLUSIONS

The causes of breast cancer remain unclear. Established risk factors include age, race/ethnicity, reproductive factors, and obesity. Unfortunately, there are few modifiable factors. Dietary factors have been implicated in the etiology of breast cancer and soy/isoflavone has been a candidate for dietary intervention. However, existing evidence from epidemiological studies is not conclusive and there have been few prospective studies of the issue. Estimation of soy and isoflavone intake, like other dietary components, is subject to measurement error. In addition, the existence of hidden sources of soy makes it difficult to estimate total isoflavone intake accurately, especially among “nonconsumers” of soy-based foods.

There is a need for more, methodologically sound, prospective studies—with extensive exposure measurement. To interpret the data, we need to consider the factors described above, namely, isoflavone dose, forms and sources of isoflavone, timing of isoflavone exposure, and the equol-producer status, estrogen-receptor status, and hormonal profile of individuals, as these are all factors that potentially modulate the association between soy intake and breast cancer risk. Other dietary, environmental, and genetic factors may also modify the association. Future studies need to address these questions by including samples large enough to detect the factors that are capable of modifying the associations between soy and breast cancer risk.
